# Intraspecific geographical variability of *Phlebotomus perniciosus* assessed by MALDI-TOF MS protein profiling

**DOI:** 10.1186/s13071-026-07354-9

**Published:** 2026-03-23

**Authors:** Vit Dvorak, Carla Maia, Rafael Marmé, José Risueño, Pedro Pérez-Cutillas, Eduardo Berriatua, Julie Sevila, Nalia Mekarnia, Jorian Prudhomme, Fano José Randriananambinintsoa, Jérôme Depaquit, Ilaria Bernardini, Claudia Mangiapelo, Gioia Bongiorno, Vladimir Ivović, Katja Adam, Petr Halada

**Affiliations:** 1 Department of Parasitology, Faculty of Science, Charles University, Prague, Czech Republic; 2 Global Health and Tropical Medicine (GHTM), LA-REAL, Instituto de Higiene E Medicina Tropical (IHMT), Universidade NOVA de Lisboa, Lisbon, Portugal; 3 Facultad de Veterinaria, Departamento de Sanidad Animal, Universidad de Murcia, Campus de Espinardo, Regional Campus of International Excellence ‘Campus Mare Nostrum’, Espinardo, Murcia Spain; 4 Facultad de Letras, Departamento de Geografía, Universidad de Murcia, Campus de La Merced, Murcia, Spain; 5 Unité Des Virus Emergents (UVE: Aix-Marseille Univ, Universita Di Corsica, IRD 190, Inserm 1207, IRBA), Marseille, France; 6 Université de Reims Champagne-Ardenne, UR ESCAPE, ANSES USC PETARD, Reims, France; 7 Laboratoire de Parasitologie-Mycologie, Pôle de Biologie Territoriale, CHU de Reims, Reims, France; 8 Department of Infectious Diseases, Vector-Borne Diseases Unit, Istituto Superiore di Sanità, Rome, Italy; 9 Faculty of Mathematics, Natural Sciences and Information Technologies, University of Primorska, Koper, Slovenia; 10 BioCeV, Institute of Microbiology of the Czech Academy of Sciences, Vestec, Czech Republic

**Keywords:** Sand fly, Intraspecific variability, MALDI-TOF mass spectrometry, Mediterranean basin, *Phlebotomus perniciosus*, Protein profiling

## Abstract

**Background:**

Matrix-assisted laser desorption/ionization time-of-flight mass spectrometry (MALDI-TOF MS) protein profiling has emerged over the last decade as a method of choice for species identification of many medically important arthropods. However, the influence of intraspecific variability on the performance of this popular technique has seldom been tested. This study provides the first standardized comparison of different geographical populations of *Phlebotomus perniciosus*, a vector of *Leishmania infantum* and Toscana virus in the western Mediterranean, by MALDI-TOF MS protein profiling.

**Methods:**

*Phlebotomus perniciosus* males were collected in five countries (Portugal, Spain, France, Italy, Croatia) that represent most of its distribution in Europe. All samples were trapped, stored and processed according to a highly standardized protocol to avoid effects other than geographical origin on their protein spectra acquired by MALDI-TOF MS protein profiling. The obtained protein spectra were compared with laboratory-reared specimens of *Ph. perniciosus*.

**Results:**

Twenty-two analysed specimens from five geographical populations provided protein spectra that were highly similar, species-specific and clustering according to their quality. No grouping according to geographical origin was observed, and the protein spectra of field-collected specimens showed similar composition and complexity to spectra from *Ph. perniciosus* laboratory colony-reared in captivity for several decades.

**Conclusions:**

Our findings demonstrate that in samples of a same sex, with the same collection method and storage time, MALDI-TOF MS protein profiling does not reflect the geographical origin of analysed specimens, confirming the value of this technique for high-fidelity and reproducible species identification of sand flies regardless of their geographical origin.

**Graphical Abstract:**

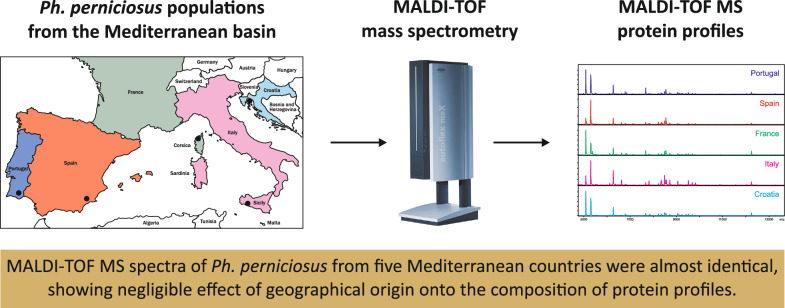

**Supplementary Information:**

The online version contains supplementary material available at 10.1186/s13071-026-07354-9.

## Background

Phlebotomine sand flies (Diptera: Psychodidae) are hematophagous insects of high importance due to biting nuisance, but more importantly as vectors of various pathogens (viruses, bacteria and parasitic protists) and disease agents with impacts in human and veterinary medicine [1]. Human leishmaniases, regarded as neglected diseases, infect 700,000–1,000,000 new individuals annually and impose a high burden in affected regions. Their negative impact is further exacerbated by poverty, social and political unrest, human migration and ongoing climatic and environmental changes, placing them among the most important human infectious diseases [2].

Transmission cycles of leishmaniases vary and may involve different sand fly vectors in different geographical regions, even for a single *Leishmania* species. Therefore, it is of paramount importance to accurately identify species that constitute local sand fly fauna, especially in endemic areas, to unravel the vectors that are implicated in pathogen transmission. However, species identification of sand flies by means of classical morphological analysis poses a serious challenge: they are of minute size, and the decisive species-specific characters require laborious dissection, mounting and microscopic inspection that necessitates expertise and experience. Therefore, molecular techniques have been warmly embraced by medical entomologists as more affordable for routine and high-throughput species identifications. In addition to a vast array of DNA-based techniques including DNA barcoding [3], protein profiling using matrix-assisted laser desorption/ionization time-of-flight mass spectrometry (MALDI-TOF MS) has become a widely used method for species identification of many medically relevant arthropods, due to its time-, labour- and cost-effectiveness that is reflected in facile and inexpensive sample preparation and analysis within a time frame of minutes to hours, depending on the number of analysed samples [4].

The method was successfully tested on sand flies, allowing conclusive species identification of both adults and immature stages [5, 6]. These proof-of-concept studies paved the way for its increasing employment for routine species identification in field studies [7–12], as a useful tool for integrative taxonomy [13–15] and eventually one of the methods for characterizing newly described sand fly species [16–18]. Despite a growing number of successful applications of MALDI-TOF MS protein profiling in sand fly studies, little attention has been devoted so far to a potential influence of intraspecific variability on the composition of protein spectra and the discriminatory power of the method to distinguish specific geographical populations within a single species. A sole study comparing specimens of several Mediterranean sand fly species, mainly of the subgenus *Larroussius*, which included specimens of geographically distant populations for some of the species tested, failed to show clear clustering based on geographical origin. Moreover, it struggled with partial confusion between *Phlebotomus perfiliewi* and *Phlebotomus perniciosus* [19]. However, these results may reflect prolonged and varying time of storage before acquisition of spectra. In this study, we aimed to assess the potential effect of intraspecific variability on protein profiles of *Ph. perniciosus*. We focused on this species of the subgenus *Larroussius* as it is a proven vector of *Leishmania infantum*, a causative agent of both human and canine leishmaniasis in the western part of the Mediterranean basin, as well as Toscana virus, which causes influenza-like syndrome and summer meningitis. To truly reflect only the species identity and geographical origin and minimize all other possible effects (trapping method, sex, storage time) on their protein spectra, a highly standardized set of samples composed of males from five European countries (Portugal, Spain, France, Italy, Croatia) was collected within a short time frame by a standardized and coordinated trapping effort within the field survey of the CLIMOS [Climate Monitoring and Decision Support Framework for Sand Fly-borne Diseases Detection and Mitigation] project (Horizon Europe, project no. 101057690, www.climos-project.eu) and subjected to a single analysis by MALDI-TOF MS protein profiling.

## Methods

### Sand fly collection

Sand flies were collected over a period of 2 months (mid-August to mid-October) at the end of the 2024 sand fly season in five countries (one locality per country, Table 1) using US Centers for Disease Control and Prevention (CDC) miniature light traps with fine collection bags (John W. Hock Company, Gainesville, FL, USA) set at dusk and operating until dawn, when live specimens were collected by aspirators, immediately placed into 70% ethanol of molecular-grade purity and shipped. After delivery, all males were inspected to confirm correct species identification: the last two abdominal segments with external genitalia were mounted using CMCP-10 mounting medium (Polysciences, Inc., Warrington, PA, USA), and decisive morphological features were observed using the identification key [20]. Subsequently, corresponding thoraxes were subjected to protein profiling analysis. In parallel, three males from our laboratory colony of *Ph. perniciosus* (Spain, 1994) kept under standard breeding conditions [21] were also included in the analysis as a control for sample preparation and MALDI-TOF MS analysis steps. The medium and storage time of these laboratory-reared specimens were the same as those of the field-collected specimens.

**Table 1:** The trapping sites in surveyed countries, specimens collected and their log score value (LSV) identification scores in the MALDI-TOF MS analysis

Country	Locality	Latitude	Longitude	Specimen ID	LSV
Portugal	Vermelhos	37°20′14.03″N	8°0′55.42″W	PL01	2.52
				PL02	2.63
				PL03	2.43
				PL04	1.78
				PL05	2.47
Spain	Lorca	37°40′36.10″N	1°41′25.14″W	SL01	2.63
				SL02	2.48
				SL03	2.38
				SL04	2.47
				SL05	2.43
France	Corte	42°33′36.99″N	8°55′53.99″E	FC01	2.45
				FC02	2.61
				FC03	2.64
				FC04	2.14
				FC05	2.43
Italy	Sciacca	37°32′26.59″N	13°9′53.05″E	IS01	2.58
				IS02	2.38
				IS03	2.48
				IS04	2.56
				IS05	2.39
Croatia	Kornic	45°2′28.96″N	14°36′52.28″E	CK01	2.53
				CK02	2.58

### MALDI-TOF MS analysis and protein spectra evaluation

Sample preparation and analysis by MALDI-TOF MS protein profiling followed a previously described protocol optimized for sand flies [5]. Thoraxes with legs and wings were manually homogenized with sterile disposable pestles in 10 μl of 25% formic acid and briefly centrifuged at 10,000×*g* for 15 s. Two microlitres of the homogenate was mixed with 2 μl of freshly prepared MALDI matrix, which was an aqueous 60% acetonitrile/0.3% trifluoroacetic acid (TFA) solution of sinapinic acid (30 mg/ml, Bruker Daltonics, Bremen, Germany). One microlitre of the mixture was then spotted on a steel MALDI plate in duplicate to avoid a possible bad crystallization on the MALDI plate. The raw spectrum was measured for both replicates, and the spectrum of higher quality selected by visual inspection and based on the spectrum intensity was recorded and further used for the analysis. Protein profiles were measured in a mass range of 3–25 kDa on an AutoFlex Speed MALDI-TOF spectrometer (Bruker Daltonics) as a sum of 10,000 laser shots (50 × 200 shots from different positions of the sample spot). The quality control and calibration of the spectrometer was performed using the Bruker Bacterial Test Standard. In total, 22 field-collected specimens (Table 1) were analysed together with three laboratory-reared specimens.

The protein spectra were visualized and compared using FlexAnalysis 3.3 software. For cluster analysis and species identification, the spectra were processed by MALDI Biotyper 3.1 software and searched against an in-house reference database comprising reference main spectrum (MSP) protein profiles of 41 different arthropod species (genera *Phlebotomus* *n* = 28; *Sergentomyia* *n* = 9; *Aedes* *n* = 2; *Culex* *n* = 1; *Lutzomyia* *n* = 1). A log score value (LSV) > 2.0 was considered as a threshold for reliable species identification. The dendrogram was created from individual MSPs of each specimen using a hierarchical clustering with correlation distance measure and average linkage. For the clustering evaluation, the quality of the obtained spectra was assessed based on the LSV identification score.

## Results

**Fig. 1:**
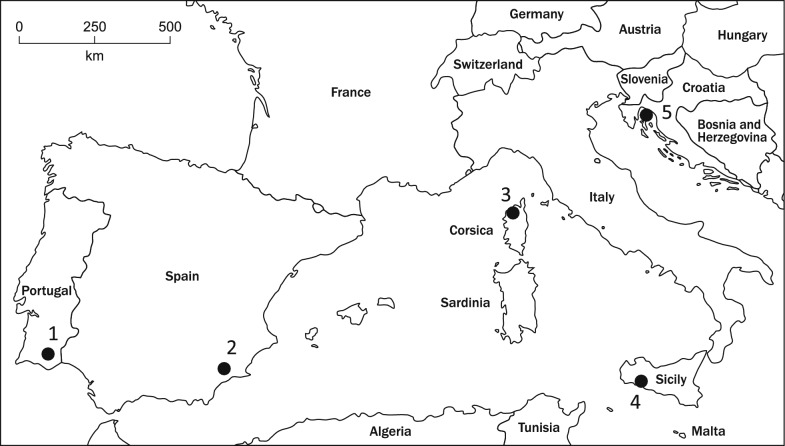
Map of the Mediterranean basin showing the sampling sites in five European countries. 1—Vermelhos (POR), 2—Lorca (ESP), 3—Corte (FRA), 4—Sciacca (ITL), 5—Kornic (CRO). The map was adapted from https://d-maps.com

**Fig. 2:**
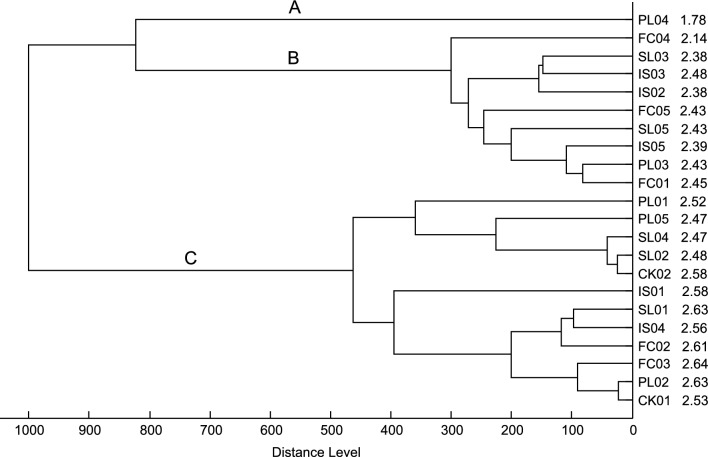
Cluster analysis of MALDI-TOF MS protein spectra of *Ph. perniciosus* specimens collected in five Mediterranean countries. Sample coding: PL—Portugal, SL—Spain, FC—France, IS—Italy, CK—Croatia. The identification LSV is shown for each sample; the distances in the dendrogram are displayed in relative units. The three branches observed may be explained by the diverse quality of the spectra judged by LSV (A: LSV = 1.78, B: mean LSV = 2.39, C: mean LSV = 2.56)

Altogether 22 sand flies (Table 1) were collected in five different countries (Fig. 1): Portugal (5), Spain (5), France (5), Italy (5) and Croatia (2). Except for one (PL04), all specimens provided protein profiles allowing unambiguous assignment of *Ph. perniciosus* species identity with LSV higher than 2.0. The obtained spectra were nearly identical and did not reveal any differences related to the geographical origin of the analysed *Ph. perniciosus* specimens (Additional file 1: Figure S1). In addition, no effect of intraspecific geographical variability was observed in the dendrogram generated by cluster analysis (Fig. 2). Interestingly, the obvious grouping may be explained by the diverse quality of the obtained spectra assessed using the LSV identification score, resulting in three branches (A: LSV = 1.78, B: mean LSV = 2.39, C: mean LSV = 2.56). The protein profiles of *Ph. perniciosus* males from the laboratory colony provided spectra of comparable quality, intensity and composition to those of field-collected specimens and clustered within the three branches depending on their LSVs (data not shown).

## Discussion

Over the last 15 years, we have witnessed growing interest in routine use of MALDI-TOF MS protein profiling for species identification of medically important arthropods as a pragmatic technique overcoming many of the shortcomings related to the traditional morphological approach or DNA-based molecular methods [4]. Beyond the proofs of concept that currently include most medically important arthropod groups (bed bugs, biting midges, fleas, horse flies, lice, mosquitoes, sand flies, ticks, triatomines and tsetse flies), subsequent studies have addressed the influence of variables such as sex, trophic status, storage regimes and time [5, 22, 23], trapping method employed [24] or body part used for the sample preparation [25] on spectrum composition.

Interestingly, the potential effect of intraspecific variability remains largely untested. Considering geographical scale, applications of MALDI-TOF MS protein profiling for species identification of arthropod vectors often represent localized studies in individual foci of a vector-borne disease outbreak [26] or field surveillance within several provinces/regions [18, 25]. A broader scope that would cover geographically distant populations of a vector species and thus depict its potential intraspecific variability across larger regions remains scarce. This is understandable, as an appropriate design of such a study poses significant methodological, logistic and budgetary challenges. To minimize bias by effects of prolonged storage, ambient conditions during shipment and varying trapping and specimen processing protocols, a highly standardized, well-coordinated, collaborative and multinational effort is needed to collate a homogeneous set of samples across different territories within an ideally short time frame—a goal difficult to achieve. Therefore, for example, while some of the mosquito species are notoriously invasive and widely distributed across continents, no data are available regarding how the protein profiling of *Aedes aegypti* or *Aedes albopictus* would reflect the structure of geographically distant populations. Generally, data on the effect of intraspecific variability on protein spectra remain scarce and give mixed results. Specifically in arthropod vectors, several studies that address this issue are discussed further.

A relatively large-scale comparison of *Ixodes ricinus* tick populations across different regions of Germany showed a structuring that may partially reflect the geographical origin. However, the authors note that the sampling localities also differed in their environmental parameters. Fluctuations in seasonal factors (humidity, temperature) may have a stronger impact on the spectrum characteristics than region of origin [27]. A comparison of two *Ae. aegypti* and two *Aedes polynesiensis* colonies, both established from different atolls of French Polynesia, made it possible to distinguish the colony of origin [28]. It is, however, problematic to draw conclusions regarding the ability to equally distinguish wild-caught specimens of the same species, as these were not included in the study. Moreover, as the colonies were of different ages, the changes in protein spectra may reflect rather the bottleneck effect that could have affected the composition of their spectra. Previously, a difference between the spectra of specimens reared in the laboratory and those of corresponding field specimens of the same species was noted for *Anopheles arabiensis* populations in Madagascar [29]; however, relatively low numbers of specimens were analysed and from only a few different geographical regions. A study aiming to distinguish the two human-biting bedbugs *Cimex hemipterus* and *Cimex lectularius* successfully identified both species [30]. It failed to differentiate wild and laboratory strains of *C. lectularius* but showed clustering by origin for *C. hemipterus*. However, only two populations were analysed (Kenya and Senegal, five specimens from each)—one wild population, the other a laboratory strain—and each was processed differently (wild specimens frozen and shipped, laboratory-reared specimens analysed fresh). Therefore, the clustering may reflect these differing parameters more than geographical origin. Protein spectrum heterogeneity of *An. arabiensis* and *Anopheles gambiae* populations from several African countries suggested an effect of geographical origin and argued for inclusion of geographically relevant spectra in the reference databases to obtain more conclusive identification [31]. Again, these results might have been impacted rather by varying preservation methods and different storage time of some analysed specimens. On the contrary, another study indicated negligible effects of geographical origin when specimens of *Ae. albopictus* from Madagascar were successfully identified by a reference database constructed using protein spectra of specimens of the same species originating in Asian (Laos, Cambodia) and Pacific (Fiji, Wallis and Futuna) regions [32]. To conclude, while some studies on medically important arthropods mentioned above show possible effects of intraspecific variability and different geographical origin on the performance of MALDI-TOF protein profiling, it may often be attributed rather to limited geographical coverage [18, 25, 26, 29], varying environmental parameters [27] and different protocols for sample acquisition, storage and processing [28–32]. This is probably a general feature, as some studies on distantly related organisms, i.e. marine copepods [33], that claim geographical clustering of protein profiles within the same species also cite the potential effect of environmental parameters or ambient conditions of sample preparation.

In our study, MALDI-TOF MS protein profiling was used to analyse specimens of *Ph. perniciosus*, one of the principal vectors of human-infecting sand fly-borne pathogens in the western Mediterranean, originating from five European countries that stretch from the western to the eastern edge of its distribution: Portugal, Spain, France, Italy and Croatia. With the exception of Slovenia, where the species is widely present [34] but from which we failed to obtain specimens for analysis within the necessary time frame due to logistical constraints, small populations of the species in southern regions of Switzerland [35] and a presumably vanished population in southern Germany where a recent field study failed to confirm the presence of the species [36], the sample set covered all European countries where the species is currently known to be distributed. To avoid the bias induced by varying collection methods, ambient storage time and medium, which compromise the previous studies as discussed above, all samples were obtained for the first time in a highly standardized manner during sand fly sampling for the CLIMOS project and analysed on a same mass spectrometer in a single run and the shortest time possible after their collection in the field. Since the project does not include partners from Maghreb countries, populations of the species from northern Africa could not be added to our analysis, as it was not possible to obtain specimens in a similar standardized fashion, which was regarded as crucial for the comparability of the spectra. Within the analysed sample set, the recorded protein spectra appeared almost identical, showed no structuring according to the geographical origin, and grouped rather according to their quality. Especially convincing is the similarity of protein spectra of specimens from Corsica and Sicily, as these originate from island populations with expected lower genetic exchange with mainland populations. The inclusion of specimens from a long-term laboratory-bred colony of *Ph. perniciosus* from Spain enabled us to assess a potential decrease in protein spectra variability, as the colony was established more than 30 years ago. However, we obtained spectra of comparable complexity, and no reduction in peak number was observed. These results, while suggesting that MALDI-TOF MS protein profiling may not be well suited for studying intraspecific variability, in fact further confirm its recently acquired position as a method of choice for reliable, time- and cost-effective high-throughput species identification of medically important arthropod vectors across their geographical distribution.

## Conclusions

This study provides results of the first rigorous and highly standardized comparison of different geographical populations of a medically important arthropod vector species, namely *Ph. perniciosus*, one of the key European sand fly vectors of *L. infantum* and Toscana virus in the western Mediterranean, by MALDI-TOF protein profiling. Benefiting from a unique opportunity to utilize the orchestrated entomological field surveys conducted in five countries within the frame of the CLIMOS project, we provide first experimental data suggesting a negligible effect of geographical origin on the composition of protein spectra, allowing the method to successfully and unambiguously assess species identity of analysed specimens. These findings further confirm the value of MALDI-TOF protein profiling as a suitable and effective method for species identification of medically important arthropods and emphasize the need to establish standardized protocols to unify specimen acquisition and storage that may have a more profound impact on its accurate performance. The results of our study, a first of its kind, shall be further corroborated by analysis of additional sand fly species and other arthropod vectors, preferably adhering to a similarly standardized approach.

## Supplementary Information

Additional file 1. Comparison of MALDI-TOF MS protein profiles of *Ph. perniciosus* sand flies collected in five European countries. One spectrum example was selected for each country. POR - Portugal, ESP - Spain, FRA - France, ITL - Italy, CRO - Croatia.

## Data Availability

All the data are presented in the main manuscript and supplementary data.
