# Supplementary figures and images for: Intraspecific geographical variability of Phlebotomus perniciosus assessed by MALDI-TOF MS protein profiling
Source: Parasit Vectors. 2026 Mar 23;19:197. doi: 10.1186/s13071-026-07354-9 (PMC13134273; doi:10.1186/s13071-026-07354-9)

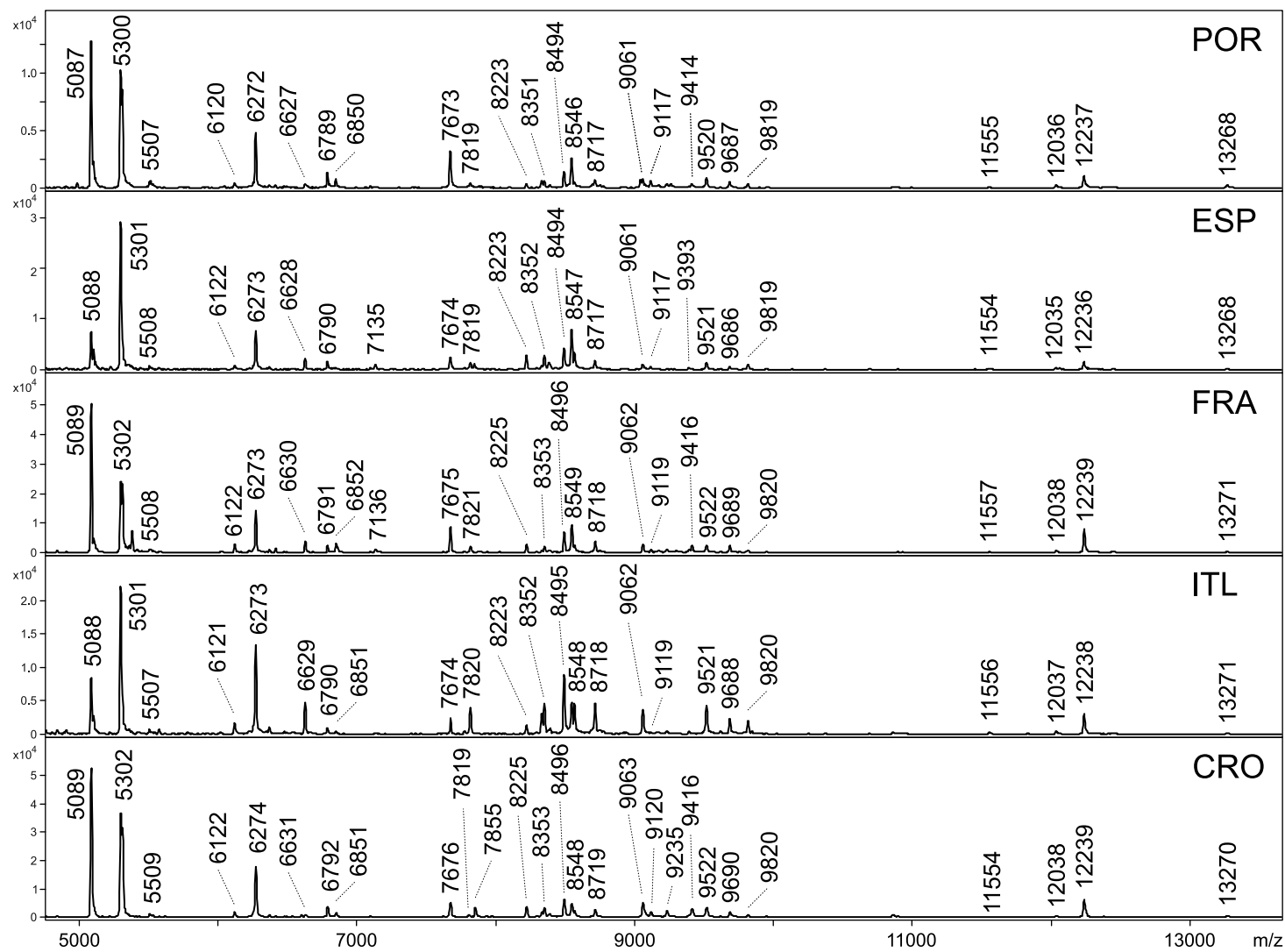

Supplement: Supplementary file 1 — Additional file 1. Comparison of MALDI-TOF MS protein profiles of Ph. perniciosus sand flies collected in five European countries. One spectrum example was selected for each country. POR - Portugal, ESP - Spain, FRA - France, ITL - Italy, CRO - Croatia. [file 13071_2026_7354_MOESM1_ESM.pdf]
